# Emphysematous changes in pneumoperitoneum and tension pneumothorax following robot-assisted bronchoscopy: a case report

**DOI:** 10.1093/jscr/rjad732

**Published:** 2024-01-18

**Authors:** Richard Q Vuong, Shawn T Liechty, Michael D Nicoara

**Affiliations:** Undergraduate Medical Education, Larner College of Medicine at the University of Vermont, Burlington, VT 05405, United States; Department of General Surgery, Danbury Hospital, Danbury, CT 06810, United States; Department of General Surgery, Danbury Hospital, Danbury, CT 06810, United States

**Keywords:** pneumoperitoneum, fiberoptic bronchoscopy, subcutaneous emphysema, pneumothorax, robot-assisted, case report

## Abstract

Pneumoperitoneum is most commonly caused by perforation of a hollow viscus but can also result as an extension of pneumothorax and/or pneumomediastinum. We present a case of pneumoperitoneum preceded by intraprocedural hemoptysis and tension pneumothorax that developed during transbronchial needle aspiration using robot-assisted flexible bronchoscopy. After stabilization and management of the pneumothorax, diagnostic laparoscopy was performed and revealed no evidence of diaphragmatic or intra-abdominal perforation but showed diffuse emphysematous changes in the gastrohepatic ligament, small and large bowel mesentery, and preperitoneal space. These findings suggest the implication of subserosal and preperitoneal emphysema as the pathophysiological mechanism of pneumoperitoneum and pneumothorax complicating bronchoscopy procedures.

## Introduction

Bronchoscopy is a commonly used diagnostic and therapeutic procedure known for its overall safety and low incidence of complications. However, like any medical intervention, it carries inherent risks, and various adverse events have been reported [1]. Pneumoperitoneum, the presence of free air in the peritoneal cavity, is a rare but potentially serious complication of bronchoscopy that is often associated with pneumothorax and/or pneumomediastinum. Only nine cases of pneumoperitoneum complicating bronchoscopy have been reported since 2003 [2]. Despite its infrequency, the precise pathophysiological mechanism behind this complication remains poorly understood. This case report aims to shed light on the involvement of emphysematous changes in the development of pneumoperitoneum and tension pneumothorax following bronchoscopy, even in the absence of perforated viscera.

## Case report

A 78-year-old female with a medical history of chronic obstructive pulmonary disease, former smoker, interstitial lung disease, coronary artery disease, hypertension, gastric ulcers and prior lung cancer of the left lower lobe treated with stereotactic radiosurgery presented with a new 0.9-cm-spiculated solid nodule of the superior segment of the right lower lobe (RLL) on a screening CT scan. Subsequent PET scan indicated increased 18-fluoro-2-deoxyglucose uptake in the nodule, consistent with malignancy. The patient was scheduled for robotic-assisted flexible bronchoscopy with biopsy and transbronchial needle aspiration (TBNA).

**Figure 1:**
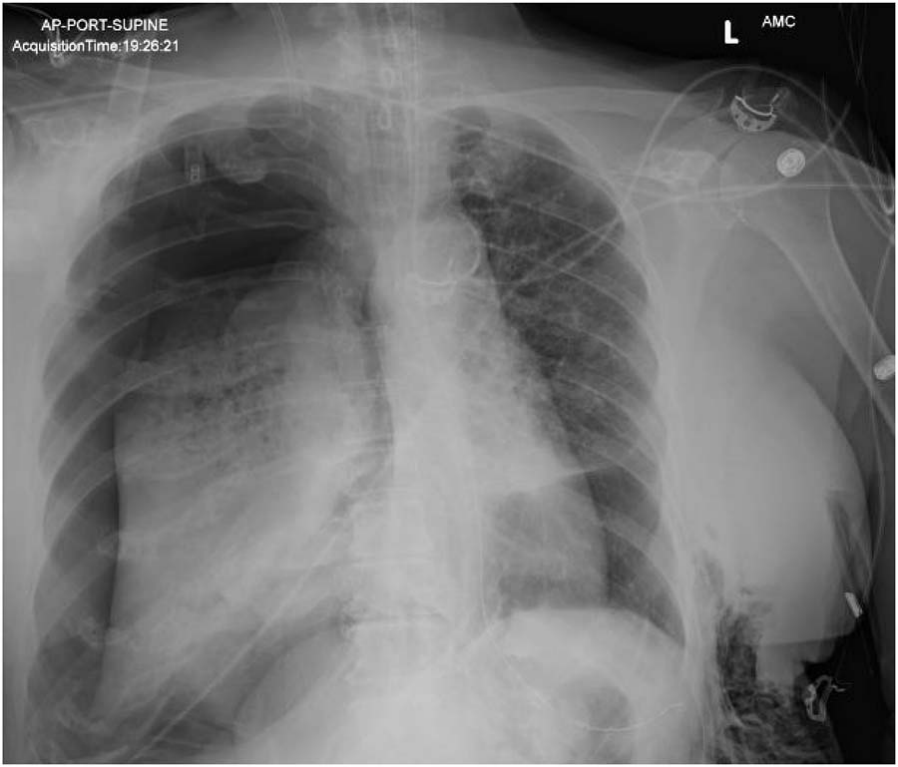
AP supine chest radiograph showing right-sided pneumothorax.

**Figure 2:**
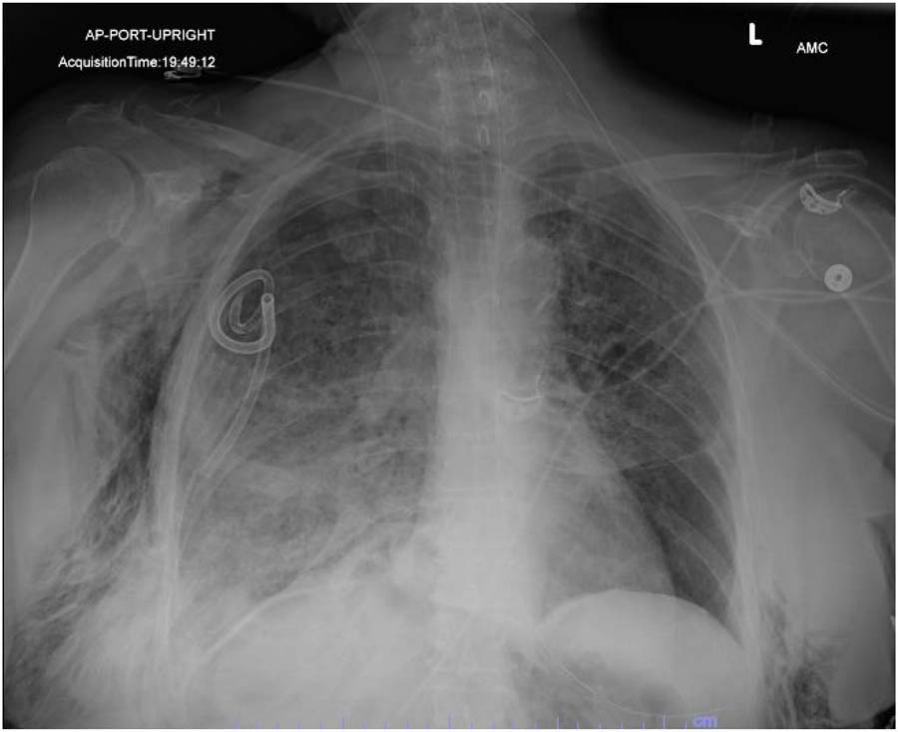
AP upright chest radiograph showing placement of right-sided chest tube and resolution of right pneumothorax.

In the operating room (OR), the patient underwent intubation for TBNA using the Monarch® robotic bronchoscopy platform. Nine needle aspirations were obtained from the third-order RLL bronchi with each aliquot subjected to rapid on-site examination. While awaiting results, the patient developed retching, hemoptysis, bronchial bleeding from the superior segment of the RLL lobe and hypotension. The bleeding was managed with ice-cold saline and epinephrine. The patient required aggressive resuscitation with crystalloid, blood products and vasopressor therapy. She was transferred to the ICU where she remained intubated for ongoing resuscitation. In the ICU, the patient developed recurrent hypotension. Chest X-ray (CXR) revealed a right-sided pneumothorax (Fig. 1) that was treated with a small-bore pigtail chest tube and resulted in immediate improvement of her tension physiology. The pneumothorax resolved on repeat chest film while the patient remained intubated and sedated (Fig. 2).

**Figure 3:**
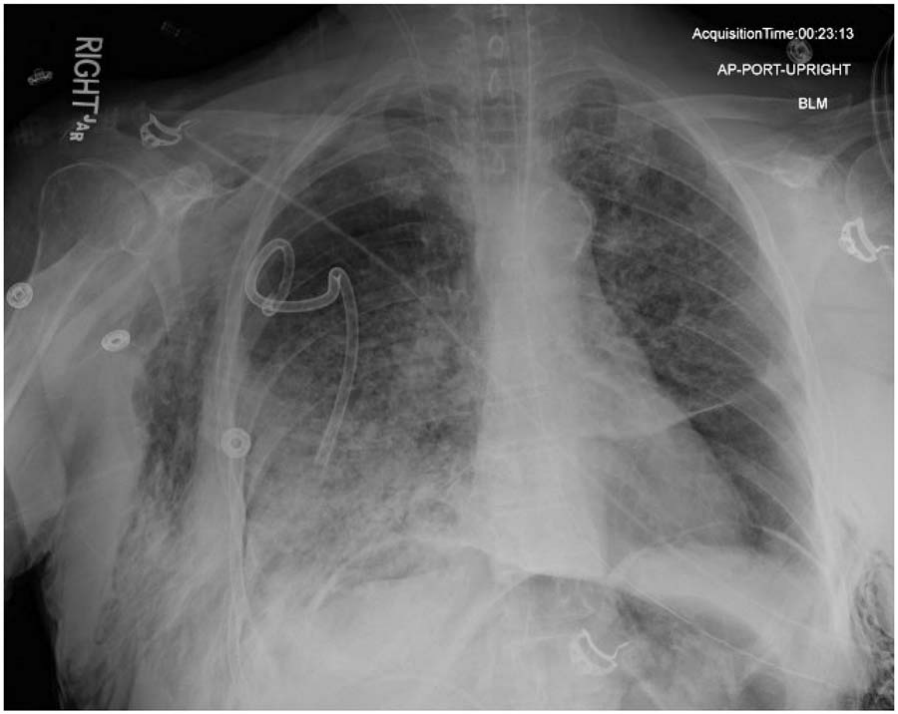
AP upright chest radiograph showing intraabdominal free air, right-sided chest tube in place, and bilateral chest wall subcutaneous emphysema that is worse on the right. Bilateral patchy airspace disease is noted as well.

**Figure 4:**
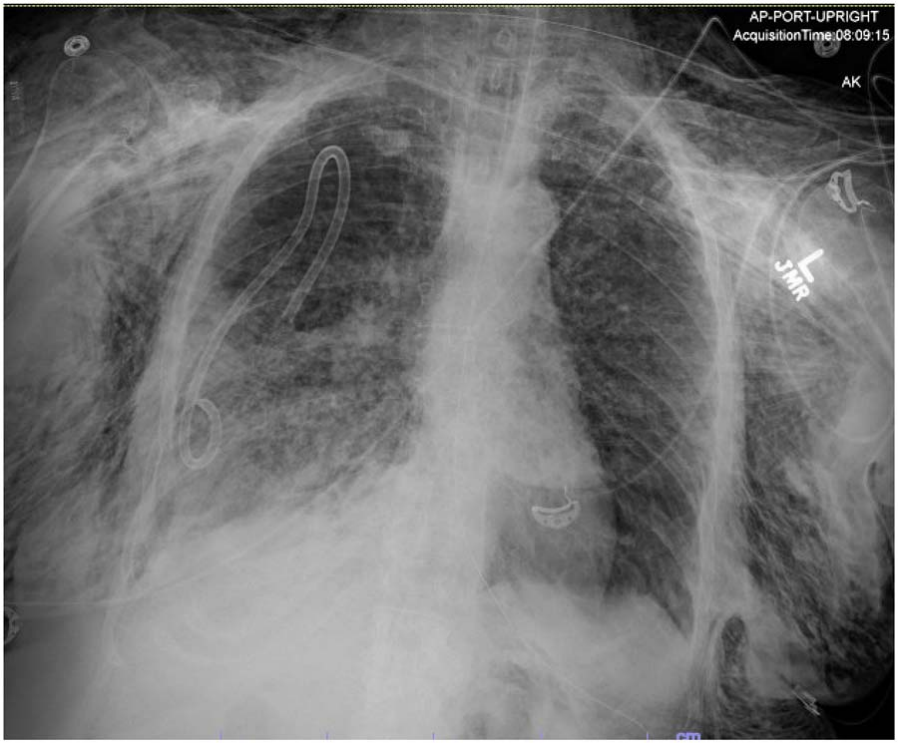
Post-operative chest radiograph showing increased diffuse subcutaneous emphysema in chest, lower neck and upper left abdominal wall. Previously demonstrated pneumoperitoneum is not visualized on the current film.

**Figure 5:**
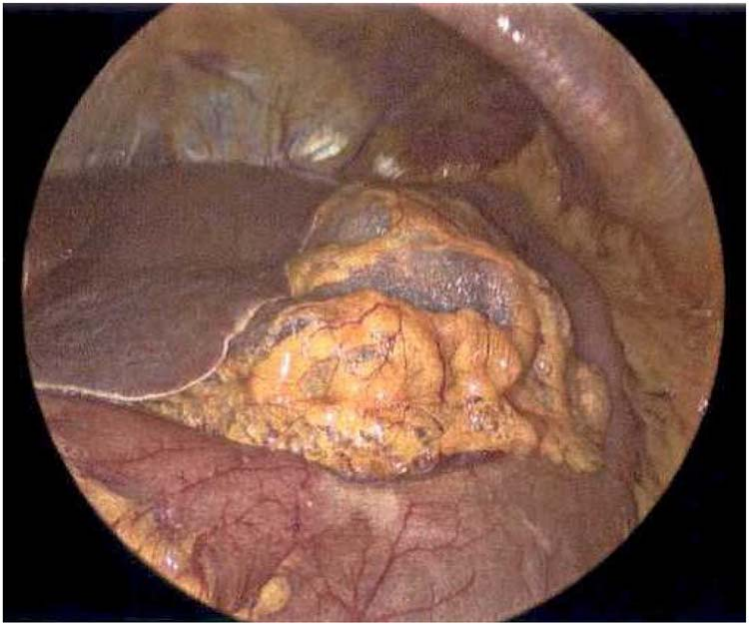
Laparoscope revealing emphysematous changes within the gastrohepatic ligament.

**Figure 6:**
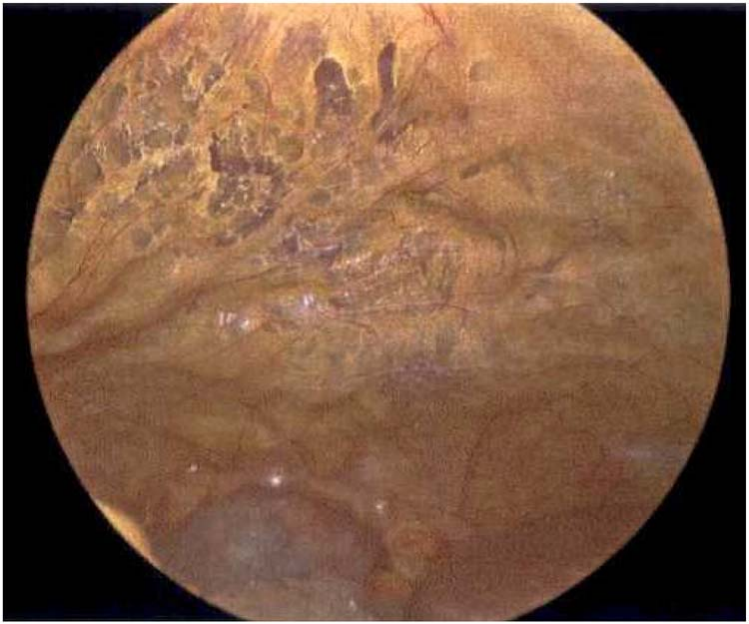
Laparoscope of the anterior abdominal wall revealing emphysematous changes within the preperitoneal space.

**Figure 7:**
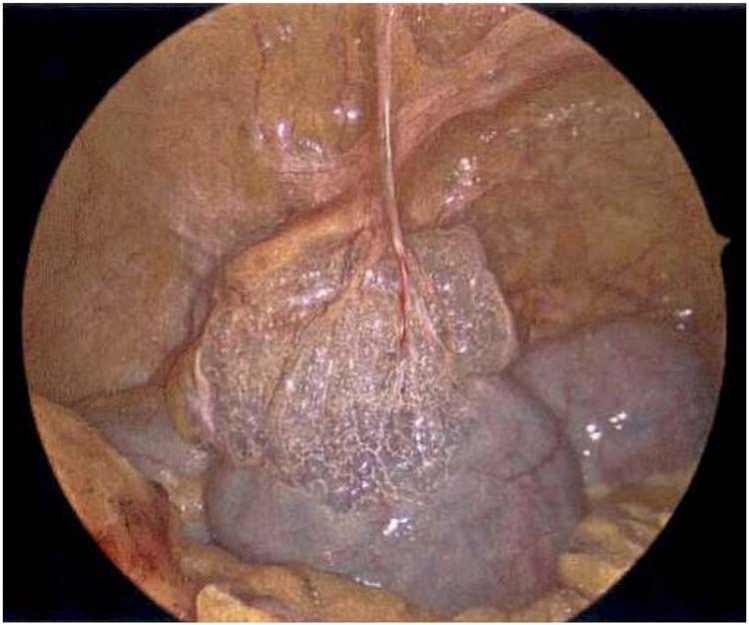
Laparoscope of the large bowel revealing subserosal emphysema within the adventitia.

**Figure 8:**
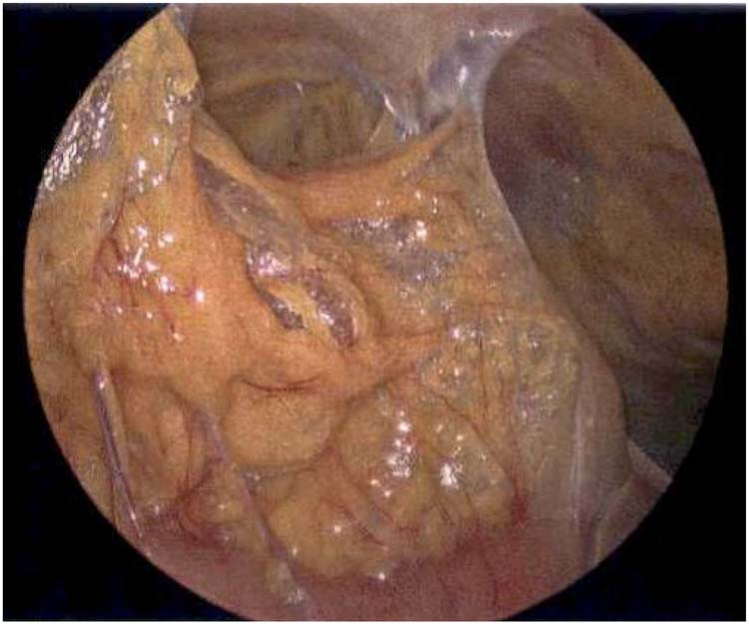
Laparoscope of the right paracolic gutter revealing emphysematous changes of the mesentery.

On Hospital Day 2, an interval CXR showed continued resolution of the right-sided pneumothorax but worsening subcutaneous emphysema of the bilateral chest wall and pneumoperitoneum (Fig. 3). Surgical consultation was sought, leading to diagnostic laparoscopy. In the abdomen, emphysematous changes within the gastrohepatic ligament and omental adhesions to the anterior abdominal wall were visualized (Figs 5 and 6). Laparoscopic exploration revealed no diaphragmatic injury or perforated viscera but identified emphysematous changes throughout the preperitoneal space, small and large bowel mesentery, and right paracolic gutter (Figs 7 and 8). Immediate post-op CXR no longer showed evidence of pneumoperitoneum (Fig. 4). The patient was transferred back to the ICU and extubated after three days on Day 5. After the removal of chest tube and transfer to a medical floor on Day 8, the patient developed aspiration pneumonia that was treated with antibiotics. She was discharged on Day 15.

## Discussion

Pneumoperitoneum is a rare complication following bronchoscopy, occurring less frequently than hemoptysis and pneumothorax, with reported cases of 0.45 and 0.10%, respectively [1]. While the immediate concern with free air in the abdomen is a perforated hollow viscus, the pathophysiology of pneumoperitoneum in the absence of visceral perforation remains unclear. No evidence of gastrointestinal perforation was found in our case, but diffuse emphysematous changes were identified throughout the abdominal cavity, suggesting a mechanism of air tracking through fascial and peritoneal planes. Past case reports have explained that pneumoperitoneum associated with pneumothorax and/or pneumomediastinum is often due to the passage of air along perivascular connective tissue or through the three diaphragmatic orifices (IVC, esophagus and aorta) [2, 3]. Others suggest diaphragmatic defects allow air to traverse from the pleura to peritoneal space [2, 4], but no diaphragmatic injury was visualized in this case. Additionally, immediate post-surgical CXR no longer showed free air in the abdomen.

The ‘Macklin’ effect may offer an explanation where alveoli under high-pressure rupture and allow free air to dissect along the bronchovascular sheaths toward the mediastinum followed by the cervical fascial planes to ultimately produce emphysematous changes that track into the abdomen [5]. This may elucidate the observed preperitoneal emphysema as a result of tracking through the preperitoneal layers via the diaphragmatic hiatus. The mechanisms causing preperitoneal emphysema were likely exacerbated by prolonged positive pressure mechanical ventilation and the retching associated with hemoptysis, which was not seen in the nine prior case reports [2]. Notably, our case provides the first intra-abdominal laparoscopic pictures of pneumoperitoneum complicating bronchoscopy.

Robotic-assisted bronchoscopy introduces a unique aspect to our case. Small prospective studies show a low incidence of pneumothorax complicating robot bronchoscopy at rates of 3.3 [6] and 3.7% [7] with an even smaller percentage requiring thoracic drainage (0.4 and 1.9%, respectively). These percentages, although higher than non-robotic bronchoscopy (1.18%) [1], may be influenced by the small sample sizes and lack of robust data from these currently ongoing trials for robotic bronchoscopy [6]. Although not described in the literature, pneumoperitoneum arising from robotic-assisted bronchoscopy raises additional concern for diaphragmatic and esophageal injury due to mechanical forces applied to the bronchoscope.

Managing pneumoperitoneum following bronchoscopy is not well described. A previous treatment protocol [2] describes the management of isolated pneumoperitoneum but is less clear on pneumoperitoneum associated with pneumothorax or pneumomediastinum and mechanical ventilation. The authors concluded that proceeding to surgical treatment or maintaining careful observation with serial abdominal exams relies on clinical signs of cardiopulmonary instability, peritonitis or relevant risk factors [2]. In this case, we were unable to assess for peritonitis due to sedation requirements and the patient had normal vital signs. However, the worsening subcutaneous emphysema, use of robotic bronchoscopy platform, history of gastric ulcers, and presence of hematemesis were concerning for diaphragmatic injury or visceral perforation. These factors influenced our decision to proceed to the OR despite hemodynamic stability. We do agree, however, in the absence of these risk factors, careful observation with serial abdominal examination is a warranted management strategy. Regardless, more reports and discussion are needed to clarify the management of pneumoperitoneum complicating bronchoscopy.
